# Cytisine for smoking cessation: A 40-day treatment with an induction period

**DOI:** 10.18332/tpc/187556

**Published:** 2024-05-27

**Authors:** Biagio Tinghino, Salvatore Cardellicchio, Flavia Corso, Chiara Cresci, Victoria Pittelli, Rosastella Principe, Licia Siracu-sano, Giovanni Zelano, Vincenzo Zagà, Maria Sofia Cattaruzza

**Affiliations:** 1 Territorial Socio-Health District of Vimercate, ASST Brianza, Italy; 2 Careggi University Hospital, Florence, Italy; 3 Addictions Department of Verona, Verona, Italy; 4 UPMC Salvator Mundi International Hospital, Rome, Italy; 5 Humanitas Research Hospital, Rozzano, Milan, Italy; 6 Servizio Dipendenze ASST di Lecco, Lecco, Italy; 7 Bologna Local Health, Bologna, Italy; 8 Sapienza University of Rome, Rome, Italy

**Keywords:** smoking cessation, nicotine, tobacco smoke, cytisine, varenicline

## Abstract

**INTRODUCTION:**

Cytisine is a smoking cessation drug now used worldwide. Most of the data available in the literature predict a 25-day treatment, accepted on the basis of previous clinical experience in Eastern Europe. There are few studies on dosing, and only recently some researchers have tried a longer treatment period.

**METHODS:**

This real-world retrospective cross-sectional study analyzed data collected consecutively from 2015 to 2021, in seven smoking cessation centers in north-central Italy. The aim of this study is to evaluate the effectiveness and tolerability of a 40-day cytisine treatment with an induction phase and a slower reduction schedule. Data were collected from a group of 871 patients treated with cysteine, varenicline, and nicotine replacement therapy (NRT). The sample was not randomized. Behavioral support (4–6 sessions, each lasting 20–30 min, plus the evaluation session) was delivered to all patients.

**RESULTS:**

Subgroups taking cytisine (n=543 for 40 days), varenicline (n=281 for 12 weeks), and NRT (n=47 for eight weeks) showed biochemically confirmed smoking abstinence at 6 months of 50.5%, 55.9%, and 51.0%, respectively, with a statistically significant difference between cytisine versus varenicline (p<0.01) but not between cytisine versus NRT (p=0.5597). Adverse events were 4.4% with cytisine and 33.3% with varenicline. Behavioral support was an important factor in effectiveness.

**CONCLUSIONS:**

This study produced preliminary evidence that the 40-day regimen of cytisine, appears to have less effectiveness in comparison to varenicline but the magnitude of the effect is comparable. The results and tolerability seem to be better than in most other studies.

## INTRODUCTION

Cytisine is a smoking cessation drug, the history of which is an interesting example of a drug derived from popular use and, at the same time, a paradox concerning registration procedure. It has been used since 1964 in Bulgaria and marketed by Sopharma Pharmaceuticals^1^. It is an alkaloid extracted from *Cytisus laburnum* (a widespread plant in Europe) used for several decades in Eastern Europe in the treatment of tobacco addiction. During World War II, cytisus leaves were used as ‘false tobacco’ when it was not possible to find traditional tobacco. This attests to the empirical finding that cytisine is able to bind brain nicotine receptors. Indeed, it has been found that cytisine is a partial agonist of the α4β2 nicotinic receptors, which has a 7-fold higher affinity for nicotine receptors, has a half-life of about 4.8 h, and is mainly eliminated by the kidneys^2-6^. Several studies have shown efficacy^7-14^ and a comparable cost-effectiveness ratio versus other molecules approved for smoking cessation. Despite this, there are persistent difficulties in obtaining cytisine registrations in European countries due to the time-consuming and costly regulatory process. Moreover, since cytisine is a natural alkaloid, the commercial interest of big companies is rather low. There is, therefore, a paradox: there are very few laboratory studies and a lot of clinical experience studies on the use of cytisine. The latter have shown great efficacy and few side effects but a wide variability of cessation rates, probably due to several factors such as dosage, dose escalation, pharmacological treatment duration, and intensity and duration of behavioral support. These operational characteristics were based on a large number of observations from a long period of use in many regions, but they were not derived from actual dose-finding studies, which are lacking. Based only on empirical experience, the most used treatment is 1.5 mg six times daily since the beginning of treatment, with a duration of 25 days and with the designated quit date scheduled on the 5th day^10,14,15^. Recently, longer durations of cytisine treatments were evaluated. In a 3-group, double-blind, placebo-controlled, randomized trial comparing two durations of cytisine treatment, 6 and 12 weeks, versus placebo, the authors used 3 mg three times daily and reported for the 6-week course of cytisine versus placebo, a continuous abstinence rate of 8.9% in weeks 3 to 24, and the 12-week course a continuous abstinence rate of 21.1% in weeks 9 to 24^16^.

In Italy, the drug has only been available as a galenic preparation for relatively few years. Its use has spread to various Italian regions with a fairly homogeneous methodology adopted by the smoking cessation centers, which followed the ‘40-day treatment’ recommended as the best practice by the Italian Society of Tobacco (SITAB)^17,18^.

The aim of this study is to retrospectively analyze the effectiveness and safety of cytisine treatment according to the ‘40-day treatment’ for smoking cessation.

## METHODS

This real-world retrospective cross-sectional study analyzed data collected consecutively from 2015 to 2021 in seven smoking cessation centers in north-central Italy, which used smoking cessation drugs as best practices recommended by the Italian Society of Tobacco (SITAB). Drugs used for smoking cessation were varenicline, nicotine replacement therapy (NRT), and cytisine according to the ‘40-day treatment’^17,18^. This last practice, published in 2015, is based on taking a 1.5 mg tablet of cytisine according to the schedule illustrated in Table 1: A gradual induction during the first week with 2 to 6 tablets per day, a maintenance week with six tablets per day, and a gradual reduction in the number of pills for the next 26 days.

**Table 1:** The 40-day treatment schedule used in seven smoking cessation centers that participated in the study

*Day*	*Tablets/day*	*Frequency of intake*
1	2	1 tab every 12 hours (8 a.m. – 8 p.m.)
2	3	1 tab every 6 hours (8 a.m. – 2 p.m. – 8 p.m.)
3	4	1 tab every 4 hours (8 a.m. –12 p.m. – 4 p.m. – 8 p.m.)
4–7	5	1 tab every 3 hours (8 a.m. – 11a.m.– 2 p.m. – 5 p.m. – 8 p.m.)
8–14	6	1 tab every 2.5 hours (8 a.m. – 10:30 a.m. – 1 p.m. – 3:30 p.m. – 6 p.m. – 8:30 p.m.)
15–21	5	1 tab every 3 hours (8 a.m. – 11 a.m. – 2 p.m. – 5 p.m. – 8 p.m.)
22–28	4	1 tab every 4 hours (8 a.m. – 12 p.m. – 4 p.m. – 8 p.m.)
29–35	3	1 tab every 6 hours (8 a.m. – 2 p.m. – 8 p.m.)
36–40	2	1 tab every 12 hours (8 a.m. – 8 p.m.)

The model is also integrated with behavioral support. The model is based on the hypothesis that gradually increasing the dose until maximum efficacy is reached may be more beneficial for both the patient (gradual adaptation to the maximum dose) and the physician (assessment of any early adverse reactions). In addition, a longer treatment period provides protection against short-term relapses, which are much more frequent in the first 30 days of therapy.

The drug was prescribed in a galenic formulation prepared in pharmacy laboratories. According to the Agenzia Italiana del FArmaco (AIFA), galenic preparations for known drugs in Europe do not require adherence to specific protocols, except adherence to good practices outlined by European standards^19^.

In each smoking cessation center, patients were not randomized to drug treatments but followed according to clinical practice. A detailed information document on cytisine was delivered, and a careful medical history and consent to data processing were collected for each patient. A tobacco use history, with exhaled carbon monoxide (CO) measurement and Fagerström test of nicotine dependence (FTND), was collected, and motivation to quit was assessed. A psychological history was carried out to evaluate the presence of anxiety, depression, use of psychotropic drugs, eating disorders, and addiction to alcohol, drugs, and gambling in the last five years.

Patients aged ≤18 years, with major decompensated psychiatric disorders (of DSM Axis I, such as severe depression and schizophrenia), with severe renal or hepatic insufficiency, and pregnant women, were excluded.

All centers adopted an integrated approach, with pharmacological and behavioral treatments lasting about eight weeks. After the first visit, a cessation-program with 4 to 6 individual, face-to-face sessions were scheduled. During each 20–30 min session, the use of the cessation drug was monitored, advice was given to cope with the difficulties encountered, behavioral techniques were used to help the patient in craving management, becoming aware of the environmental cues, and recognizing the emotions related to quit smoking.

Patients were placed in three categories according to their outcome after six months of follow-up: ‘abstinents’, ‘smokers’, and ‘drop-outs’ (interruption of treatment).

A cessation rate, confirmed by CO <10 ppm, was performed at the end of treatment and at 1, 3, and 6 months later. Patients with CO >10 ppm or missing data were counted as smokers. Self-reported adverse events were recorded.

CO-verified continuous abstinence at six months and self-reported adverse events (AEs) are the primary outcomes of this study.

### Statistical analysis

To describe sample characteristics and evaluate differences, percentages and χ^2^ tests were used for categorical variables, while means with standard deviation (SD) and t-tests were used to analyze differences among means; a p<0.05 was considered statistically significant. All analyses were performed using Stata (StataCorp. 2020. Stata Statistical Software: Release 16.1 College Station, TX: StataCorp LLC).

## RESULTS

**Figure 1:**
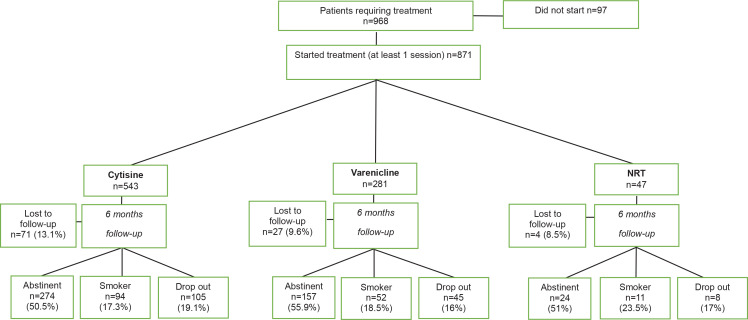
Characteristics of the patients according to treatment, success rate, and drop-ou

From a total of 968 patients who asked for an appointment for smoking cessation in the seven Italian centers that took part in this study, 97 patients did not start, and 871 attended at least one session. Data recorded for these 871 patients were retrospectively examined: 543 patients were treated with cytisine, 281 with varenicline, and 47 with NRT. Figure 1 shows the characteristics of these patients according to their treatments, success rate, and drop-out.

Sociodemographic characteristics, number of cigarettes smoked per day, exhaled CO, and Fagerström test of nicotine dependence are reported in Table 2.

**Table 2:** Characteristics by treatment group of patients treated in seven smoking cessation centers in north-central Italy, 2015–2021 (N=871)

*Characteristics*	*Cytisine (N=543) Mean (SD)*	*Varenicline (N=281) Mean (SD)*	*NRT (N=47) Mean (SD)*	Cytisine vs Varenicline p*	*Cytisine vs NRT p*
Age (years)	52.5 (11.8)	54.4 (11.5)	54.1 (13.8)	**0.0274**	0.3796
Sex (female), %	50.2	53.4	51.4	0.4403	0.9614
Cigarettes/day	21.2 (8.8)	22.0 (11.1)	16.1 (10.1)	0.2594	<0.01
Exhaled CO (ppm)	21.9 (9.4)	20.6 (8.3)	20.2 (5.0)	0.0507	0.2213
FTND score	6.0 (1.8)	5.7 (2.0)	4.7 (2.5)	**0.0294**	**<0.01**
Abstinence (yes), %	50.5	55.9	51.0	**<0.01**	0.5597

FTND: Fagerström test for nicotine dependence.

* χ^2^ test and t-test according to type of variable; in bold statistically significant p-values.

After six months of follow-up, the percentages of biochemically confirmed continuous smoking abstinence were 50.5% in the cytisine (40 days), 55.9% in the varenicline (12 weeks), and 51.0% in the NRT (8 weeks) groups, with a statistically significant difference between cytisine versus varenicline (p<0.01) but not between cytisine versus NRT (p=0.5597) (Table 2).

In the varenicline group, 58 (20.6%) patients reported an adverse event [gastric complaints n=30 (10.6%), intestinal disorders n=12 (4.2%), sleep disturbances n=7 (2.5%), other n=9 (3.2%)], while in the cytisine group only 24 (4.4%) patients reported an adverse event [gastric complaints n=8 (1.4%), headache n=5 (0.9%), sleep disturbances n=3 (0.5%), intestinal disorders n=3 (0.5%), other n=5 (0.9%)]. In the NRT group, only 3 (6.4%) adverse events were reported (n=2 local dermatitis for patch, and n=1 headache). No severe adverse events occurred in either group, but 4 and 3 patients discontinued therapy for adverse events in the cytisine and varenicline groups, respectively.

## DISCUSSION

A very recent systematic review and meta-analysis showed that cytisine increases the chances of successful smoking cessation by more than two-fold compared with placebo15. In the present study, the 6-month cessation rate in the cytisine (40 days) group, even if less effective in comparison to varenicline, showed a comparable magnitude of the effect. The reported cessation rates were 50.5%, 55.9%, and 51.0%, respectively, in the cytisine (40 days), varenicline (12 weeks), and NRT (8 weeks) groups. The effectiveness of cytisine found in our study is higher compared with other studies using cytisine with a treatment schedule of 25 days^10,14,15^ or with a recent study by Rigotti et al.^16^ (3-group, double-blind, placebo-controlled, randomized trial) who found continuous abstinence rates of 8.9% during weeks 3 to 24 in the group of 6-week duration. In the same study, the abstinence rate increased to 21.1% during weeks 9 to 24 for the 12-week course. Also, the recent study by Walker20, conducted in New Zealand on 679 Maori people randomly assigned to receive a prescription for 12 weeks of cytisine or varenicline, observed a 12.1% continuous abstinence rate at six months for cytisine versus 7.9% for varenicline. In the literature, longer cytisine treatments (in comparison to 25 days) accumulate and show greater efficacy. This is also consistent with the results observed for other drugs of proven efficacy.

Treatments with varenicline, bupropion, and NRT have shown that durations ranging from 8 to 12 weeks produce higher effectiveness. On this last consideration, several cessation treatment services in Italy adopted the 40-day treatment practice since several years^17,18^ whose data are analyzed here.

In addition to prolonging the duration of treatment, it was hypothesized (in analogy with other treatments with partial agonists, such as varenicline) that an induction period might allow for a gradual effect of the drug, a wider margin of observation of side effects, and a better adaptation by the patient to the effects of cytisine. The results of this study seem to confirm these hypotheses.

Moreover, a longer treatment might also be more effective in ensuring better extinction of craving and consolidation of cessation, providing greater protection against the period of vulnerability to relapse.

Our data suggest a similar magnitude of effectiveness for cytisine, varenicline and NRT. It is possible that our high intensity behavioral support (4 or more sessions, each lasting an average 20–30 min) may have had a favorable effect.

In our study, with regard to AEs, the group treated with cytisine tolerated the drug well and experienced low frequency and minor side effects compared to the varenicline group.

In the literature, the number of side effects associated with cytisine use is variable and higher than in our study. In 2014, Walker et al.^14^ found that cytisine caused 288 AEs in 204 participants with a ratio of 1.41. In the study by Tindle et al.^21^, AEs in the cytisine group were 31 events reported by 86 participants with a ratio of 0.36. In a trial by Courtney et al.^22^, the AEs in the cytisine group were 997 events among 482 participants with a ratio of 2.06. In another study by Walker^20^, 313 events occurred over six months in the cytisine group in 111 participants, with a ratio of 2.80 events per patient.

In our group of cytisine-treated patients (n=543), the number of AEs was 24, with a ratio of 0.04, thus much lower than the data reported in most studies. The ratio was 0.10 for the varenicline group (n=281 patients, 30 AEs).

Some of the AEs (e.g. headache or sleep disturbance) are characteristic of tobacco abstinence and are not necessarily related to the use of the drug, but there was no placebo group in our study, so we cannot evaluate this aspect. In our experience, adverse events with cytisine were few and mild. The gradual induction period seems to be well accepted by patients, helps them gain confidence in the treatment, and monitors the possible onset of side effects. It is possible that the ‘long’ induction period affects the lower number of adverse events. We hypothesize that progressive receptor saturation may reduce side effects compared with a shorter schedule (i.e. 25 days).

### Limitations

This study has numerous limitations. There was no placebo group, and there was no direct comparison with the 25-day scheme. However, the comparison between these two modalities was formulated on the basis of other studies in the literature that were not conducted in Italy. Another limitation was that the assignment to treatment groups (cystine, varenicline, and NRT) was not randomized, and the NRT group was very small. The choice between a partial agonist drug (varenicline or cystine) and NRT was driven in the first instance by patients’ preferences. At the beginning of the observed period, when varenicline and cytisine were both available, the choice between them was expressed by the patient mainly according to economic reasons, as the cost of the galenic preparation of cytisine for the entire treatment was much lower than that for varenicline (about 60 vs 325 euros for varenicline). This factor has an important impact in Italy, as treatments are not reimbursed, except for varenicline (but only for a few categories of subjects). Subsequently, the choice between varenicline and cytisine was influenced by external factors, including the fact that varenicline in 2021 was no longer available in Italy. For these reasons, it is possible to speculate that the choice of treatments was little influenced by clinical evaluations.

Beyond these limitations, this study does provide preliminary evidence that the 40-day regimen of cytisine appears to have high effectiveness and good tolerability in the real world.

## CONCLUSIONS

To our knowledge, this is the first study that evaluates the effectiveness and tolerability of a novel regimen of cytisine: a 40-day treatment with an induction phase and a slower reduction schedule.

The results about cytisine use show higher biochemically confirmed continuous smoking abstinence and fewer self-reported side effects compared to most studies. This study produced preliminary evidence that cystine, albeit with the many limitations of the present study, appears to have a comparable magnitude of effectiveness to varenicline and NRT. Moreover, behavioral support may be a contributing factor in explaining the high cessation rate achieved. Further studies are needed to evaluate the best duration of treatment, possible different intensities, and types of behavioral support.

## Data Availability

The data supporting this research are available from the authors on reasonable request.
